# PTEN-Regulated AID Transcription in Germinal Center B Cells Is Essential for the Class-Switch Recombination and IgG Antibody Responses

**DOI:** 10.3389/fimmu.2018.00371

**Published:** 2018-02-28

**Authors:** Jing Wang, Sichen Liu, Baidong Hou, Meixiang Yang, Zhongjun Dong, Hai Qi, Wanli Liu

**Affiliations:** ^1^MOE Key Laboratory of Protein Sciences, Collaborative Innovation Center for Diagnosis and Treatment of Infectious Diseases, School of Life Sciences, Institute for Immunology, Tsinghua University, Beijing, China; ^2^Key Laboratory of Infection and Immunity, Institute of Biophysics (CAS), Beijing, China; ^3^School of Medicine, Institute for Immunology, Tsinghua University, Beijing, China; ^4^Laboratory of Dynamic Immunobiology, Department of Basic Biomedical Sciences, School of Medicine, Institute for Immunology, Tsinghua Peking Center for Life Sciences, Tsinghua University, Beijing, China

**Keywords:** PTEN, germinal center, IgG1^+^ B cells, class-switch recombination, somatic hypermutation

## Abstract

Class-switch recombination (CSR) and somatic hypermutation (SHM) occur during the differentiation of germinal center B cells (GCBs). Activation-induced cytidine deaminase (AID) is responsible for both CSR and SHM in GCBs. Here, we show that ablation of PTEN through the Cγ1-Cre mediated recombination significantly influences the CSR and SHM responses. The GCs fail to produce the IgG1 B cells, the high affinity antibodies and nearly lost the dark zone (DZ) in *Pten^fl/fl^Cγ1^Cre/+^* mice after immunization, suggesting the impaired GC structure. Further mechanistic investigations show that LPS- and interleukin-4 stimulation induced the transcription of Cγ1 in IgM-BCR expressing B cells, which efficiently disrupts PTEN transcription, results in the hyperphosphorylated AKT and FoxO1 and in turn the suppression of AID transcription. Additionally, the reduced transcription of PTEN and AID is also validated by investigating the IgM-BCR expressing GCBs from *Pten^fl/fl^Cγ1^Cre/+^* mice upon immunization. In conclusion, PTEN regulated AID transcription in GCBs is essential for the CSR and IgG antibody responses.

## Introduction

The germinal center (GC) is the region where antigen-activated B cells undergo proliferation and differentiation responses to differentiate into either plasma cells or memory B cells. Somatic hypermutation (SHM) and class-switch recombination (CSR) occur during the proliferation of GC B cells (GCBs) (1–3). SHM and CSR account for the generation of high affinity and class-switched B cells in humoral immunity (4). There are eight sets of C_H_ exons at the *Igh* locus in mice, which are constituted as 5′-Cμ-Cδ-Cγ3-Cγ1-Cγ2b-Cγ2a-Cε-Cα-3′. During the CSR, the assembled V(D)J exons from Cμ encoded IgM-expressing B cells is juxtaposed next to one of the sets of the downstream C_H_ exons, converting IgM-expressing B cells to different IgH sub-classes (e.g., IgG3, IgG1, and IgG2b), which are, respectively, encoded by different C_H_ genes (e.g., Cγ3, Cγ1, and Cγ2b) (5). Activation-induced cytidine deaminase (AID), as the B cell-specific factor, is required for the CSR (6). During GC responses, AID produces C:G to U:G and even C:G to A:T mismatches (7), which then triggers the mismatch and base-excision repairs. Furthermore, the generation of DNA double-strand breaks (DSBs) at switch regions between Sμ and a downstream S region leads to a rearranged C_H_ locus and the deletion of the intervening sequence (8, 9). The repair of the AID induced DSBs *via* nonhomologous end-joining (NHEJ) eventually completes the CSR by rejoining the two broken S regions (10, 11).

Previous studies suggested that the phosphatidylinositol-3-kinase (PI3K) and AKT signaling can both regulate the *Ig* gene rearrangement during B cell development and the CSR during GC responses (12–18). Phosphatase and tension homolog (PTEN) is known to negatively regulate PI3K-mediated growth, survival, proliferation and cellular metabolism of B cells (16, 17, 19–22). Thus PTEN deficiency alters B1, marginal zone B (MZB) and follicular B (FOB) cell subsets in *Pten^fl/fl^CD19-Cre* mice (16, 17). Further study revealed that imbalanced PTEN and PI3K signaling impaired the μHC recombination in pro-B cells in *Pten^fl/fl^mb1-Cre* mice (12). Recently, emerging efforts have been placed to investigate the molecular mechanism of PTEN- and PI3K-tuned AKT signaling in regulating the strength of GC responses (14, 15, 23). B cell specific deficiency of PTEN in *Pten^fl/fl^mb1^Cre/+^* mice leads to the severe defects of B cell development at the bone marrow stage due to failed VJD recombination (12). The loss of the mature naïve B cell population in *Pten^fl/fl^mb1^Cre/+^* mice prevented the assessment of the function of PTEN in GCB-mediated CSR and antibody responses. As a solution, PTEN was recently knocked out in mature B cells in *Pten^fl/fl^hCD20Tam^Cre/+^* mice, which demonstrated the importance of PTEN in regulating GC responses (23). Although mature B cell specific deficiency of PTEN in *Pten^fl/fl^hCD20Tam^Cre/+^* mice excluded the B developmental defects as in the case of *Pten^fl/fl^mb1^Cre/+^* mice, the usage of *Pten^fl/fl^hCD20Tam^Cre/+^* mice cannot explicitly separates the function of PTEN in mature B cell activation and proliferation upon antigen stimulation versus that in GC responses since GCBs were differentiated from activated mature naïve B cells after antigen stimulation. Here, to precisely assess the function of PTEN in GCB-mediated humoral responses *in vivo*, we used a mouse model with a PTEN deletion only in specific subsets of GCBs. Our results reveal that PTEN regulated AID transcription in GCBs is essential for the CSR and IgG antibody responses.

## Materials and Methods

### Mice, Cell Culture

C57BL/6J (B6) background *Pten^fl/fl^* mice (a kind gift from Dr. Wei Guo, Tsinghua University) were mated to *Cγ1-Cre* transgenic mice (a kind gift from Dr. Tomohiro Kurosaki, Osaka University and Dr. Klaus Rajewsky, Max Delbrück Center) in which expression of Cre is controlled by the endogenous promoter of the B cell-specific gene Cγ1. Offspring carrying *Cγ1-Cre* and two copies of the floxed *Pten* allele or *Cγ1-Cre* plus two copies of the WT *Pten* allele were used in the analyses as homozygous mutant (*Pten^fl/fl^Cγ1^Cre/+^*) or WT (*Pten^+/+^Cγ1^Cre/+^*) mice, respectively. All mice were maintained under specific pathogen-free conditions and used in accordance of governmental and institutional guidelines for animal welfare. Primary B cells were negatively isolated from the spleen of *Pten^+/+^Cγ1^Cre/+^* or *Pten^fl/fl^Cγ1^Cre/+^* mice as previously reported (24). Single cell suspensions were cultured in RPMI-1640 medium supplemented with 10% FBS, 50 µM β-mercaptoethanol (Sigma-Aldrich), penicillin/streptomycin antibiotics (Invitrogen) and Non-Essential Amino Acids (Invitrogen). B cells were stimulated for 4 days using 10 µg/mL LPS (Sigma) alone or LPS plus 50 ng/mL interleukin-4 (IL-4) (R&D) or 1 µg/mL anti-CD40 (eBioscience) alone or anti-CD40 plus 50 ng/mL IL-4 (R&D) in order to drive primary B cells class-switch *in vitro*.

### Immunization, ELISA Assay, and Immunohistochemistry

For mice GC Flow cytometry analysis, 6-week-old *Pten^+/+^Cγ1^Cre/+^* and *Pten^fl/fl^Cγ1^Cre/+^* mice were injected intraperitoneally with 1 × 10^9^ sheep red blood cells (SRBCs, Bioren, China) or emulsified BSA in Alum adjuvant then analysis at day 7 after the immunization. Qβ virus-like particles (VLPs) were expressed in *E. coli* strain JM109 with exogenous expression plasmid pQ10 and then purified. The CpG contained VLPs were obtained by packaging VLPs with CpG ODN G10 *in vitro* as described (25). 6-week-old *Pten^+/+^Cγ1^Cre/+^* and *Pten^fl/fl^Cγ1^Cre/+^* mice were injected intraperitoneally with 10 µg VLP in 400 µL PBS for the immunization. Mice were analyzed at day 7 or day 14 after immunization. For NP-antigen specific T-cell-dependent immunization, 6-week-old *Pten^+/+^Cγ1^Cre/+^* and *Pten^fl/fl^Cγ1^Cre/+^* mice were injected on footpad with 10 µg NP_33_-KLH in 20 µL PBS and boost at day 35. Mice were bled at the indicated days after immunization.

To detect VLP or NP-specific IgM, IgG, IgG1, IgG2b, IgG2c, and IgG3 in immunized mice (6 *Pten^+/+^Cγ1^Cre/+^* and 6 *Pten^fl/fl^Cγ1^Cre/+^*), 2 µg/mL VLP, 5 µg/mL NP_8_-BSA or 5 µg/mL NP_30_-BSA were coated on maxisorb plates (Nunc) and incubated overnight at 4°C. All these plates were blocked with 0.3% gelatin in PBS buffer (2 h at 37°C), followed by addition of pre-diluted mice serum into each well and incubated at 37°C for 1 h. 1:10,000 diluted HRP conjugated goat anti-mouse IgM, IgG, IgG1, IgG2b, IgG2c, and IgG3 were used to detect VLP or NP-specific antibodies also the innate immune antibodies. Then followed the protocol as published before for the final readout results (26).

For immunohistochemistry 6-week-old *Pten^+/+^Cγ1^Cre/+^* and *Pten^fl/fl^Cγ1^Cre/+^* mice were injected intraperitoneally with 1 × 10^9^ SRBC in PBS. Spleen sections were frozen from day 7 after immunization in OCT compound (Sakura Finetek). After that spleen sections were stained with Alexa Fluor 488 anti-mouse/human CD45R/B220 (#103225, BioLegend), PE anti-mouse FAS (#152608, BioLegend), and APC anti-mouse CD3ε (#100312, BioLegend) by following the protocol described by Sandrine Sander (14).

### Western Blotting

Primary splenic B cells or cultured cells were lysed by 2× Lysis buffer. Proteins were extracted by 10% Bis-Tris PAGE (Life technologies) with the cocktail of proteinase inhibitors (Life technologies) in it, transferred to polyvinylidene fluoride (PVDF). The electroblotted membranes were blocked by TBST containing 5% non-fat milk (BD) and were probed with anti-PTEN, anti-β-actin, anti-p-AKT 473, or anti-p-FoxO1/3a primary antibodies overnight at 4°C then immunoblotted with HRP-conjugated secondary antibodies (Dako). PTEN (138G6) Rabbit antibody (#9559), Phospho-AKT (Ser473) rabbit antibody (#4060), phospho-FoxO1 (Thr24)/FoxO3a (Thr32) rabbit antibody (#9464), and β-actin (13E5) rabbit antibody (#4970) were purchased from Cell Signaling Technology. AID rabbit antibody was a kind gift from Dr. Feilong Meng (Shanghai Institute of Biochemistry and Cell Biology, China).

### RT-PCR

Pure *Pten^+/+^Cγ1^Cre/+^* and *Pten^fl/fl^Cγ1^Cre/+^* splenic B cells were stimulated for 4 days with 10 µg/ml LPS and 50 ng/mL IL-4. Pre-incubated IgM-BCR expressing B cells were sorted by using FACSAria III Cell Sorter (BD). SRBC immunized *Pten^+/+^Cγ1^Cre/+^* and *Pten^fl/fl^Cγ1^Cre/+^* mice IgM-BCR expressing GCBs were first enriched by CL-7 marker (GL-7 monoclonal antibody, Biotin, eBioscience; streptavidin microbeads, Miltenyi Biotec) by using the LS columns (Miltenyi Biotec). On the basis of enrich, cells were sorted by using FACSAria III Cell Sorter (BD) after labeling with IgM-BCR expressing GCBs dyes (anti-CD19-APC, anti-FAS-PE, Alexa Fluor 488 goat Fab anti mouse IgM μ chain and ef450-streptavidin for GL-7).

Total RNA was extracted using TRIzol (GIBCO) according to the manufacturer’s instructions and was subjected to the reverse transcription to make cDNA. For PCR of post-switch Cγ1, transcript was amplified using the following primers pair: (Cγ1, 429 bp) 5′-GGC CCT TCC AGA TCT TTG AG-3′ (Cγ1 forward), 5′-GGA TCC AGA GTT CCA GGT CAC T-3′ (Cγ1 reverse). For amplification of the PTEN (387 bp) transcript, the primer pair of 5′-TTG AAG ACC ATA ACC CAC CAC AG-3′ (PTEN forward) and 5′-GGC AGA CCA CAA ACT GAG GAT TG-3′ (PTEN reverse) was used. Forward primer for AID (349 bp): 5′-GAG GGA GTC AAG AAA GTC ACG CTG GA-3′, reverse primer for AID: 5′-GGC TGA GGT TAG GGT TCC ATC TCA G-3′. Forward primer for control GAPDH (566 bp): 5′-TGT GTC CGT CGT GGA TCT GA-3′ and reverse primer for GAPDH: 5′-TTG CTG TTG AAG TCG CAG GAG-3′. PCR conditions were 94°C for 3 min, 94°C for 1 min, 60°C for 45 s, 72°C for 45 s, 30 cycles (from step 2 to step 4), and 72°C for 10 min for final extension.

### Flow Cytometry

Cells were preincubated with FcBlock (anti-CD16/32, eBiosence) to minimize nonspecific staining. Cells were then stained with cocktails of various mAbs (anti-CD19, -B220, -IgG1, -IgM, -IgD, -CD43, -CD93, -CD21, -CD23, -CD5, -FAS, -CL-7, -CD3ε, -CXCR4, or anti-CD86) conjugated with different fluoresce proteins. All antibodies were purchased from BD, eBioscience or BioLegend. Labeled cells were detected by 5 lasers Fortessa (BD), all data were analyzed with FlowJo software. Cell sorting was performed by using FACSAria III Cell Sorter (BD), following the protocols provided by BD.

### Microscopy Instruments

Confocal images were captured using Olympus FV-1000 microscope equipped with 10 × objective lens and a 405, a 473, a 549, and a 635 nm laser. The acquisition was controlled by FV10-ASW4.0 software.

### Image Process and Statistical Analyses

Images were analyzed by Image Pro Plus (Media Cybernetics) software following our previous protocols (27). Statistical tests were performed with Prism 5.0 (GraphPad). Two-tailed *t* tests were used to compare end-point means of different groups. Statistical significant results (*p*) are indicated as: *p < 0.05; **p < 0.01, and ***p < 0.001.

## Results

### Normal Development and Homeostasis of B Cells in *Pten^fl/fl^Cγ1^Cre/+^* Mice

**Figure 1:**
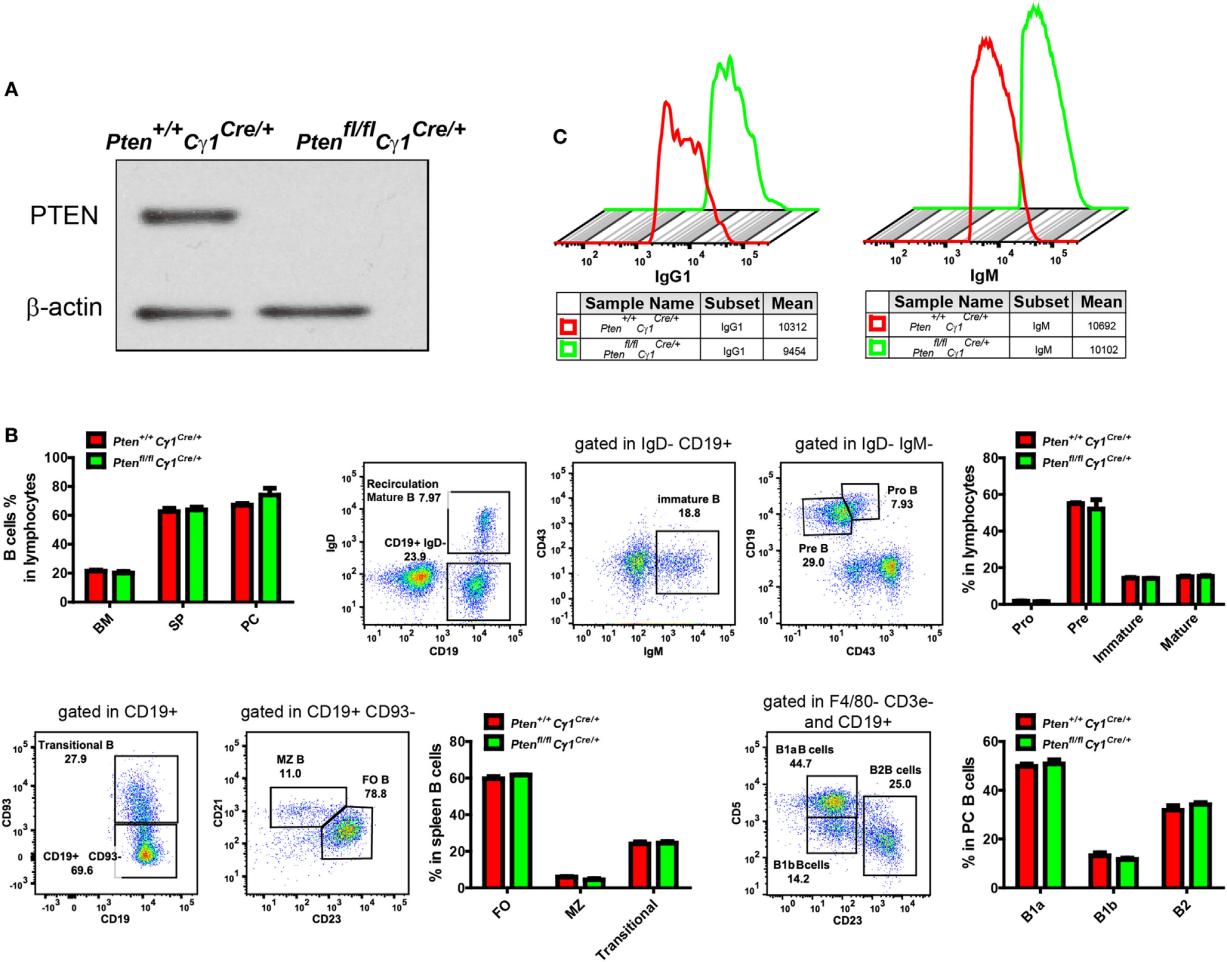
Normal development and homeostasis of B cells in *Pten^fl/fl^ Cγ1^Cre/+^* mice. **(A)** Expression of PTEN in IgG1-BCR expressing splenic B cells from *Pten^+/+^ Cγ1^Cre/+^* and *Pten^fl/fl^ Cγ1^Cre/+^*. Data were given from one representative of at least three independent experiments. **(B)** Comparable B cell percentage in bone marrow (BM), spleen (SP), and peritoneal cavity (PC) (left, top pattern). B cell development in BM (right, top pattern), SP and PC (bottom pattern). MZ, marginal zone; FO, follicular; PC, peritoneal cavity; SP, spleen. The data represent the mean ± SEM of four mice per group in three independent experiments. **(C)** Comparable amount of surface IgG1- (left) or IgM- (right) BCR level in the indicated type of B cells from *Pten^+/+^ Cγ1^Cre/+^* and *Pten^fl/fl^ Cγ1^Cre/^*mice. Data were given from one representative of at least three independent experiments.

To detect the PTEN function in GCBs, we generated the *Pten^fl/fl^Cγ1^Cre/+^* mice by breeding *Pten^fl/fl^* mice to *Cγ1-Cre* mice. In *Pten^fl/fl^Cγ1^Cre/+^* mice, PTEN would only be knocked out in B cells with Cγ1 transcription, which are ideally the IgG1-BCR expressing B cells (Figure 1A). We avoided to breed *Pten^fl/f^* mice with *Aicda^Cre/+^* mice since the *Pten^fl/fl^Aicda^Cre/+^* mice have been reported to develop severe submandibular hair loss, skin thickening and the manifestation of squamous papillomas (28).

We first examined the development and homeostasis of B cells in *Pten^fl/fl^Cγ1^Cre/+^* mice and confirmed that PTEN deletion in IgG1-BCR expressing B cells did not affect B cell development in the bone marrow and peripheral lymphoid organs (Figure 1B). Further flow cytometry analysis of the splenic IgM and IgG1-BCR expressing B cells showed comparable amounts of surface IgM or IgG1 BCRs in *Pten^fl/fl^Cγ1^Cre/+^* versus *Pten^+/+^Cγ1^Cre/+^* control mice (Figure 1C).

### Impaired Antibody Responses in *Pten^fl/fl^Cγ1^Cre/+^* Mice

**Figure 2:**
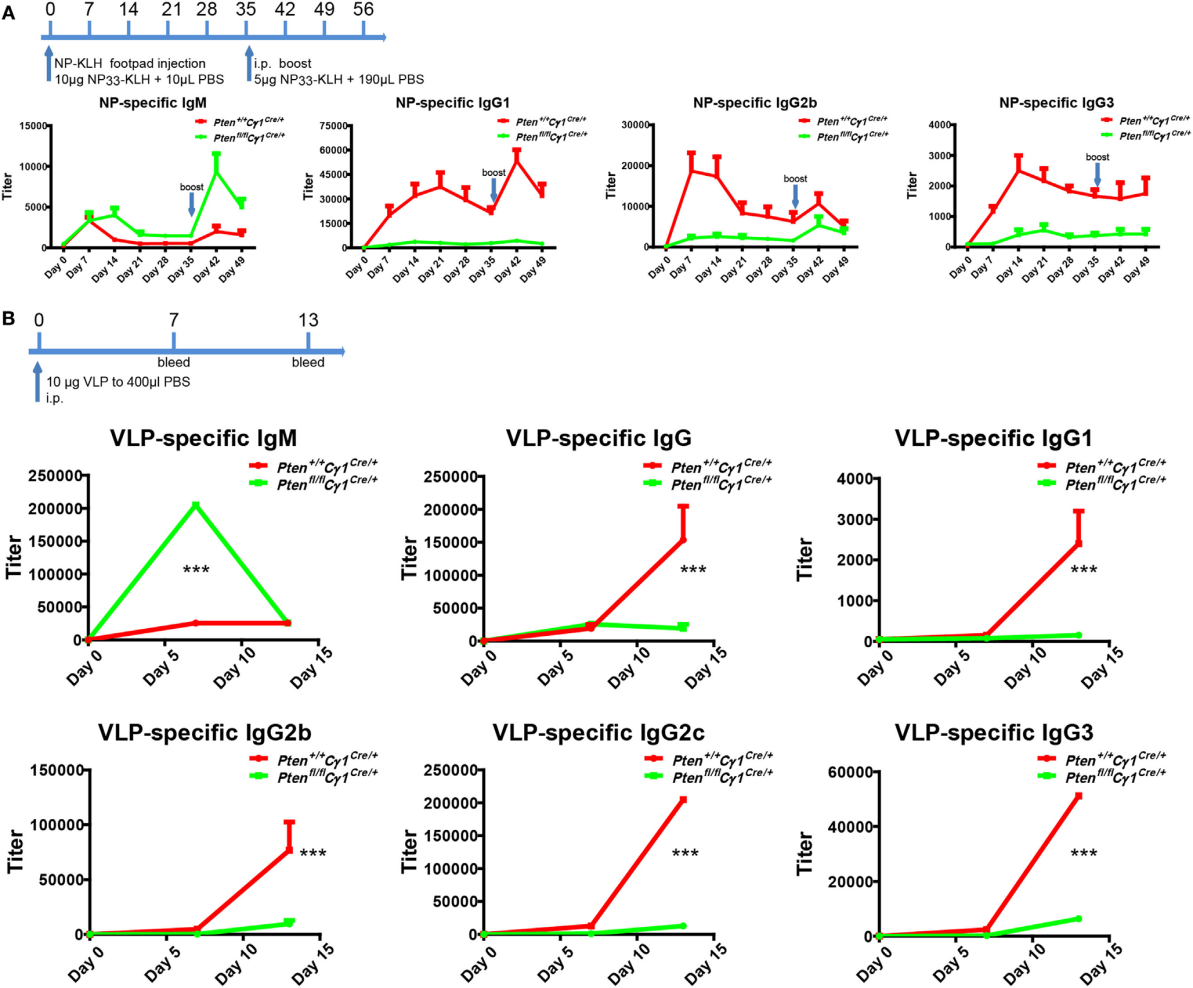
Impaired antibody responses in *Pten^fl/fl^ Cγ1^Cre/+^* mice. **(A)** Titers of NP-specific antibodies in the *Pten^+/+^ Cγ1^Cre/+^* and *Pten^fl/fl^ Cγ1^Cre/+^* mice upon immunization with 10 µg NP_33_-KLH at day 0. NP-specific antibody titers in IgM, IgG1, IgG2b, and IgG3 isotypes were examined by ELISA using NP_8_-BSA (5 µg/ml) as the coating Ag. The data represent the median ± interquartile range from three independent experiments with six mice per group at the indicated time point and were analyzed with Kruskal–Wallis test. **(B)** The production of virus-like particle (VLP)-specific antibodies in *Pten^+/+^ Cγ1^Cre/+^* and *Pten^fl/fl^ Cγ1^Cre/+^* mice upon the immunization (i.p.) with 10 µg VLP at day 0, day 7, and day 14. VLP specific antibody titers in IgM, IgG and IgG1, IgG2b, IgG2c, or IgG3 isotype were examined by ELISA experiment by using VLP (2 µg/ml) as the coating Ag. The data represent the median ± interquartile range from three independent experiments with six mice per group at the indicated time point and were analyzed with Kruskal–Wallis test. ***p < 0.001.

*Pten^fl/fl^Cγ1^Cre/+^* mice showed B cell normal development that allowed us to examine the humoral responses upon the immunization with either the T cell-dependent (TD) antigen NP_33_-KLH or the Qβ VLPs as reported (25). Age and gender matched *Pten^+/+^Cγ1^Cre/+^* and *Pten^fl/fl^Cγ1^Cre/+^* mice were undergone footpad injection with 10 µg NP_33_-KLH in 20 µL PBS and boost at day 35 for the TD antigen immunization (Figure 2A). For Qβ virus immunization, 6-week-old *Pten^+/+^Cγ1^Cre/+^* and *Pten^fl/fl^Cγ1^Cre/+^* mice were immunized intraperitoneally with 10 µg VLP in 400 µL PBS (Figure 2B). ELISA analyses showed that the IgM antibody responses upon the induction by both NP-KLH and VLP were significantly higher in the *Pten^fl/fl^Cγ1^Cre/+^* mice compared to the control *Pten^+/+^Cγ1^Cre/+^* mice (Figures 2A,B). However, the production of not only IgG1 but also IgG2b and IgG3 were significantly blunted in the *Pten^fl/fl^Cγ1^Cre/+^* mice (Figures 2A,B). We hypothesized that the decreased IgG antibody responses may be due to a damaged CSR reaction within the GC of *Pten^fl/fl^Cγ1^Cre/+^* mice.

### Damaged CSR in *Pten^fl/fl^Cγ1^Cre/+^* Mice

**Figure 3:**
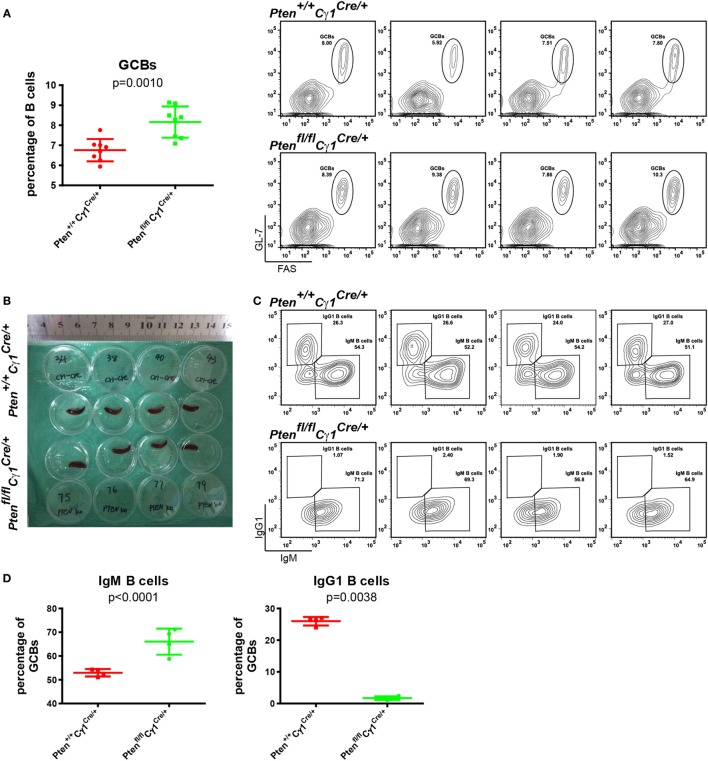
Damaged class-switch recombination in *Pten^fl/fl^ Cγ1^Cre/+^* mice. **(A)** The statistical comparison of the percentage of germinal center B cells (GCBs) from splenocytes at day 7 after sheep red blood cell (SRBC) immunization (left). Each symbol represents an individual animal (*n* = 8 for each group). The data represent the mean ± SD from at least three independent experiments. Two-tailed *t*-tests were performed for statistical comparisons. Flow cytometry analysis the increased GCBs number in the *Pten^fl/fl^ Cγ1^Cre/+^* mice spleen (right). Values indicated the percentage of cells in the gated population. **(B)** Macroscopic appearance of spleen from day 7 after SRBC immunization of *Pten^+/+^ Cγ1^Cre/+^* and *Pten^fl/fl^ Cγ1^Cre/+^* mice. Data were given from one representative of at least two independent experiments. **(C)** Representative flow cytometry analysis of IgM, IgG1-BCR expressing GCBs from *Pten^+/+^ Cγ1^Cre/+^* and *Pten^fl/fl^ Cγ1^Cre/+^* mice at day 7 after SRBC immunization. Data were given from one representative of at least two independent experiments (n = 4 for each group). The cells were pregated in GCBs. **(D)** The statistical quantification of the flow cytometry data of IgM (left) and IgG1 (right) as shown in **(C)**. Each symbol represents an individual animal (four mice for each group). The data represent the mean ± SD. Two-tailed *t*-tests were performed for statistical comparisons.

To test whether or not PTEN deletion in IgG1^+^ B cells will damage the CSR within the GC reaction, GCs were induced by the immunization of SRBC in both *Pten^fl/fl^Cγ1^Cre/+^* and *Pten^+/+^Cγ1^Cre/+^* control mice. Flow cytometry analyses of the splenic B cells at day 7 after SRBC injection unexpectedly demonstrated an increased but not decreased levels of GCBs in *Pten^fl/fl^Cγ1^Cre/+^* mice than the control *Pten^+/+^Cγ1^Cre/+^* mice even though the size of the spleen of both types of mice was comparable (Figures 3A,B). Remarkably, further analyses showed that the IgM-BCR expressing GCBs were obviously increased while the class-switched IgG1-BCR expressing GCBs were almost lost in SRBC immunized *Pten^fl/fl^Cγ1^Cre/+^* mice GC (Figures 3C,D). Similar results were also obtained in the immunized *Pten^fl/fl^Cγ1^Cre/+^* and *Pten^+/+^Cγ1^Cre/+^* mice by the utilization of alum adjuvant-precipitate BSA (Figures S1A,B in Supplementary Material). These results suggested that PTEN deletion in IgG1^+^ B cells significantly damaged the CSR within GCs.

To further verify the above observation of the impaired CSR within the GC of *Pten^fl/fl^Cγ1^Cre/+^* mice, we purified the splenic B cells and performed an *in vitro* CSR assay following the published protocols (13, 16). Clearly, B cells from *Pten^fl/fl^Cγ1^Cre/+^* mice failed to undergo CSR to form IgG1-BCR expressing B cells in the presence of lipopolysaccharide (LPS) alone or LPS plus IL-4 after 4 days of stimulation, respectively (Figures S2A,B in Supplementary Material). Similar results were acquired in the presence of other CSR-driven reagents of anti-CD40 antibodies or anti-CD40 plus IL-4 (Figures S2A,B in Supplementary Material).

### Abnormal GC Structure and SHM in *Pten^fl/fl^Cγ1^Cre/+^* Mice

**Figure 4:**
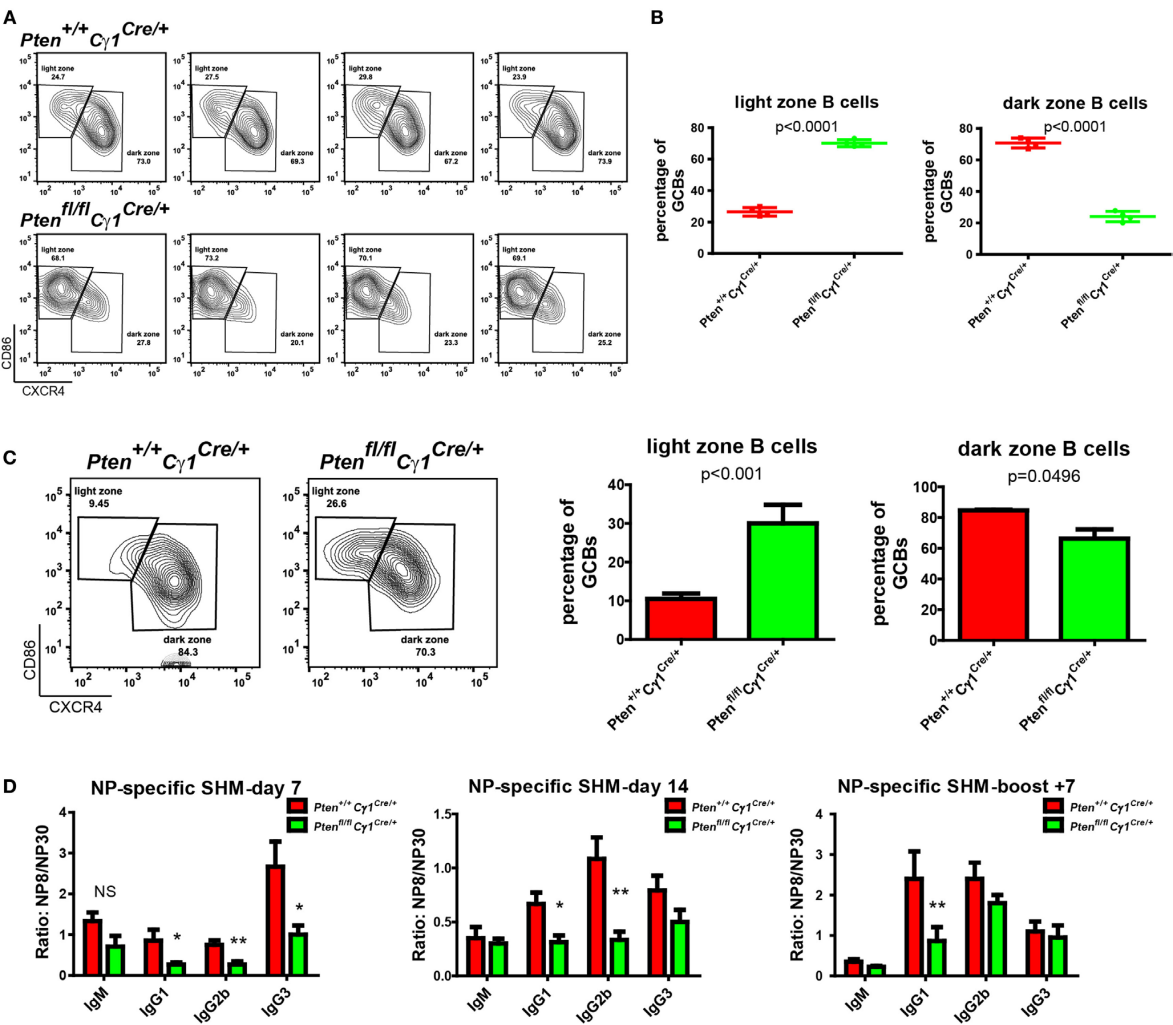
Abnormal germinal center (GC) structure and somatic hypermutation (SHM) in *Pten^fl/fl^ Cγ1^Cre/+^* mice. **(A)** Representative flow cytometry analysis of GC light zone (LZ) and dark zone (DZ) splenocytes from *Pten^+/+^ Cγ1^Cre/+^* and *Pten^fl/fl^ Cγ1^Cre/+^* mice at day 7 after SRBC immunization. Data were given from one representative of at least two independent experiments (*n* = 4 for each group). The cells were pregated in GCBs. **(B)** The statistical quantification of the flow cytometry data of LZ (left) and DZ (right) splenocytes as shown in **(A)**. Each symbol represents an individual animal (four mice for each group). The data represent the mean ± SD. Two-tailed *t*-tests were performed for statistical comparisons. **(C)** Representative flow cytometry analysis of GC LZ and DZ from *Pten^+/+^ Cγ1^Cre/+^* and *Pten^fl/fl^ Cγ1^Cre/+^* mice at day 14 after VLP injection (left). Statistical comparison for LZ (middle) and DZ (right) cells percentage of total GCBs was also shown. The data represent the mean ± SD of six mice per group in three independent experiments. Two-tailed *t* tests were performed for statistical comparisons. **(D)** The affinity maturation of the NP-specific IgM, IgG1, IgG2b, and IgG3 antibodies in *Pten^+/+^ Cγ1^Cre/+^* versus *Pten^fl/fl^ Cγ1^Cre/+^* mice as determined by the ratio NP8/NP30. The data represent the median ± interquartile range from three independent experiments with six mice per group at the indicated time point and were analyzed with Kruskal–Wallis test. *p < 0.05; **p < 0.01.

**Figure 5:**
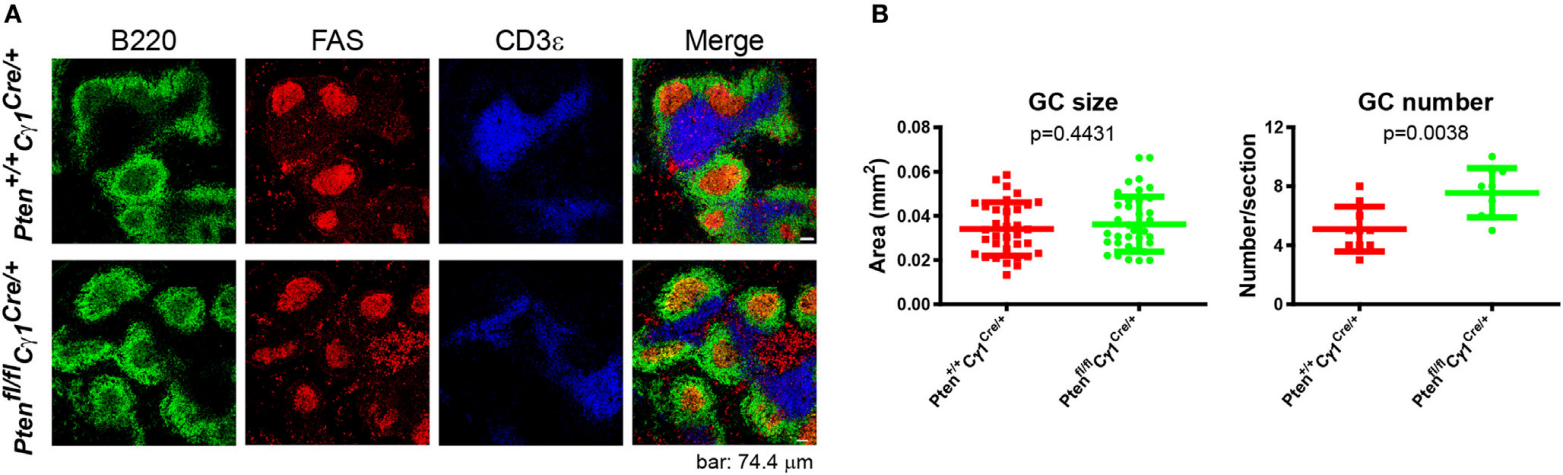
Normal germinal center (GC) size, but abnormal GC number in *Pten^fl/fl^ Cγ1^Cre/+^* mice. **(A)** Immunofluorescence analysis of spleen sections from immunized *Pten^+/+^ Cγ1^Cre/+^* and *Pten^fl/fl^ Cγ1^Cre/+^* mice at day 7 after sheep red blood cell immunization. Antibodies detecting B220 (expressed on B cells), FAS (expressed by GCBs), and CD3ε (expressed on T cells) were utilized. Data were given from one representative of at least three independent experiments. The scale bar represents 74.4 µm. **(B)** The statistical quantification of the GC size (left) measured in terms of area or the GC number (right) measured in terms of the number of GCs per each spleen section in *Pten^+/+^ Cγ1^Cre/+^* and *Pten^fl/fl^ Cγ1^Cre/+^* mice as described in **(A)**. The bars represent the mean ± SD from one representative of at least three independent experiments. Two-tailed *t* tests were performed for statistical comparisons.

It is known that GC contains the light zone (LZ) and the dark zone (DZ). GCBs undergo consecutive and cyclic phases of proliferation and SHM in the DZ, followed by the migration to the LZ, where they capture and internalize antigen for the acquisition of survival signals from follicular helper T cells (3, 29, 30). We thus quantified the formation of LZ and DZ within the GC and found that DZ and LZ compartmentalization was severely disturbed in the *Pten^fl/fl^Cγ1^Cre/+^* mice upon the immunization by both SRBC and VLP (Figures 4A–C). Moreover, we observed that the number of DZ B cells in *Pten^fl/fl^Cγ1^Cre/+^* mice GCs was dramatically decreased, consistent with a recent study showing that upon FoxO1 ablation or induction of PI3K activity, GCs lost their DZ, owing at least partly to downregulation of the chemokine receptor CXCR4 (14, 15). Since an essential step in the selection of high affinity GCBs is the recruitment of LZ GCBs into the GC DZ (31, 32), it is not a surprise that the SHM in GCBs was significantly damaged in *Pten^fl/fl^Cγ1^Cre/+^* mice upon the immunization by TD antigen NP_33_-KLH (Figure 4D). Lastly, it should be noted that even though the GC DZ formation was impaired in the *Pten^fl/fl^Cγ1^Cre/+^* mice, the size of the GCs was normal in the spleen section as detected by immunofluorescence and the number of GCs per spleen section was even higher in these PTEN KO mice (Figures 5A,B), which are consistent with the results that *Pten^fl/fl^Cγ1^Cre/+^* mice exhibited an increased levels of GCBs than the control *Pten^+/+^Cγ1^Cre/+^* mice upon immunization (Figure 3A; Figure S1B in Supplementary Material).

### Repression of AID Induction in GCBs from *Pten^fl/fl^Cγ1^Cre/+^* Mice

**Figure 6:**
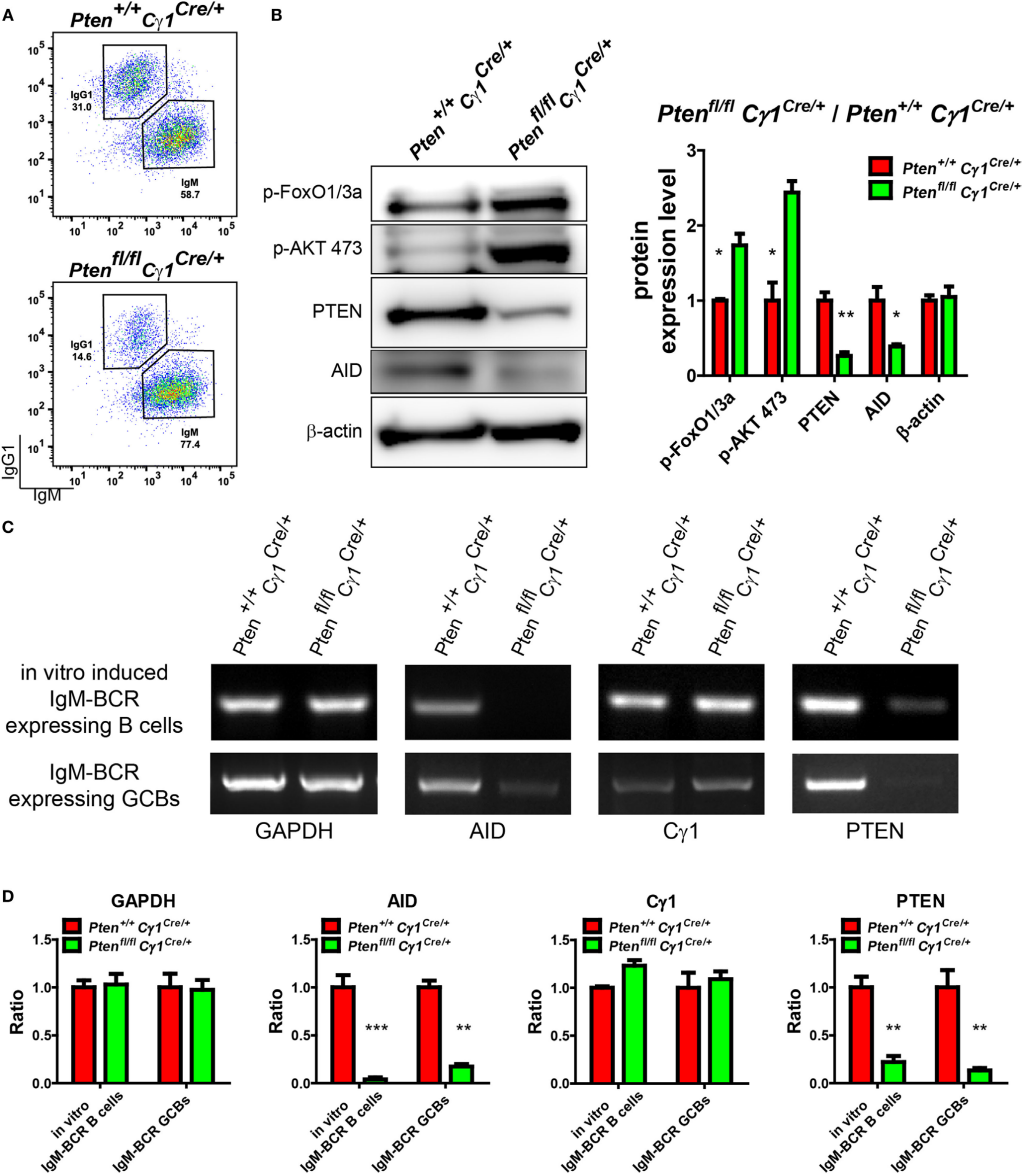
Repression of activation-induced cytidine deaminase (AID) induction in germinal center B cells (GCBs) from *Pten^fl/fl^ Cγ1^Cre/+^* mice. **(A)** Flow cytometry sorting of *in vitro* induced IgM-BCR expressing splenic B cells from *Pten^+/+^ Cγ1^Cre/+^* and *Pten^fl/fl^ Cγ1^Cre/+^* mice. Pure splenic B cells from control and KO mice (three mice for each group) were stimulated with LPS plus interleukin-4 (IL-4) for 4 days before sorting. **(B)** The expression levels of PTEN, p-AKT, p-FoxO1/3a, and AID were determined in *Pten^+/+^ Cγ1^Cre/+^* and *Pten^fl/fl^ Cγ1^Cre/+^* IgM-BCR expressing B cells (left). Reduced PTEN, AID expression and enhanced p-AKT and p-FoxO1/3a expression were observed in *Pten^fl/fl^ Cγ1^Cre/+^* IgM-BCR expressing B cells. The *in vitro* induced IgM-BCR expressing B cells were isolated by the FACSAria III Cell Sorter as described in **(A)**. Statistical comparison of protein expression level was also shown in right. Ratio values of different proteins from *Pten^fl/fl^ Cγ1^Cre/+^* IgM-BCR expressing B cells were normalized to that of the *Pten^+/+^ Cγ1^Cre/+^* IgM-BCR expressing B cells. Data were given from one representative of at least three independent experiments. *p < 0.05; **p < 0.01. **(C)** The mRNA levels of AID, Cγ1, and PTEN were analyzed by RT-PCR. The Cγ1 mRNA was detected in both *Pten^fl/fl^ Cγ1^Cre/+^* IgM-BCR expressing splenic B cells that was induced by LPS and IL-4 *in vitro* and the sheep red blood cell immunized *Pten^fl/fl^ Cγ1^Cre/+^* IgM-BCR expressing GCBs. The PTEN mRNA level was reduced and the AID mRNA expression level was nearly undetectable in *Pten^fl/fl^ Cγ1^Cre/+^* mice. The *in vitro* induced IgM-BCR expressing B cells were isolated by the FACSAria III Cell Sorter as described in **(A)**, and the IgM-BCR expressing GCBs were isolated by the FACSAria III Cell Sorter as described in (Figure S3 in Supplementary Material). Data were given from one representative of at least three independent experiments. **(D)** Statistical comparison of GAPDH, AID, Cγ1, and PTEN transcription level. Given were the ratio values of different molecules from *Pten^fl/fl^ Cγ1^Cre/+^* IgM-BCR expressing B cells to that of the *Pten^+/+^ Cγ1^Cre/+^* IgM-BCR expressing B cells or from *Pten^fl/fl^ Cγ1^Cre/+^* IgM-BCR expressing GCBs to that of the *Pten^+/+^ Cγ1^Cre/+^* IgM-BCR expressing GCBs. Data were given from one representative of at least three independent experiments. **p < 0.01; ***p < 0.001.

Our observations suggested that Cγ1 transcription-mediated PTEN KO impairs antibody responses, CSR activity, as well as the DZ and LZ compartmentalization and SHM within the GC. We next investigated the molecular mechanism accounting for the lack of CSR and SHM in the B cells from *Pten^fl/fl^Cγ1^Cre/+^* mice. It is well known that AID, which is specifically induced in the GCBs, is a crucial enzyme responsible for both CSR and SHM (6). Thus, we hypothesized that the PTEN expression level in these *Pten^fl/fl^Cγ1^Cre/+^* mice shall be influenced before the CSR reaction to drive the switch of IgM-BCR to IgG-BCR expressing B cells, which might subsequently impair the function of AID. To test this hypothesis, splenic B cells from *Pten^fl/fl^Cγ1^Cre/+^* and *Pten^+/+^Cγ1^Cre/+^* mice were stimulated with LPS and IL-4 to induce CSR *in vitro*. To specifically examine the B cells without effective CSR, we sorted the IgM-BCR expressing B cells from the LPS- and IL-4 stimulated splenic B cells (Figure 6A). WB of these stimulated IgM-BCR expressing B cells detected the reduced PTEN and AID protein expression in the cells derived from *Pten^fl/fl^Cγ1^Cre/+^* mice compared to the *Pten^+/+^Cγ1^Cre/+^* control mice (Figure 6B). Meanwhile, these IgM-BCR expressing B cells also showed hyper-phosphorylated AKT and FoxO1 (Figure 6B). These results suggested that the AID transcription was affected by the hyper-phosphorylation of AKT and FoxO1 since AKT are known to inhibit the expression and function of AID (16, 23). Indeed, RT-PCR assay demonstrated that the level of PTEN and AID mRNA was markedly reduced in the stimulated IgM-BCR expressing B cells from *Pten^fl/fl^Cγ1^Cre/+^* mice than those B cells from the *Pten^+/+^Cγ1^Cre/+^* control mice (Figure 6C, top and Figure 6D). The transcription of Cγ1 was also detected in both the murine control and KO IgM-BCR expressing B cells, which readily explained the Cγ1-mediated PTEN deletion in the IgM-BCR expressing B cells in *Pten^fl/fl^Cγ1^Cre/+^* mice (Figure 6C, top and Figure 6D). We future validated these conclusions by utilizing purified GBCs *in vivo* from SRBC immunized *Pten^+/+^Cγ1^Cre/+^* and *Pten^fl/fl^Cγ1^Cre/+^* mice. We sorted the IgM-BCR expressing GCBs at day 7 after SRBC immunization (Figure S3 in Supplementary Material). RT-PCR of IgM-BCR expressing GCBs from *Pten^fl/fl^Cγ1^Cre/+^* mice also detected the significantly reduced transcription of PTEN and markedly reduced AID (Figure 6C, bottom and Figure 6D). The transcription of Cγ1 was also detected in IgM-BCR expressing GCBs from both *Pten^+/+^Cγ1^Cre/+^* and *Pten^fl/fl^Cγ1^Cre/+^* mice (Figure 6C, bottom and Figure 6D), which readily explained the Cγ1-mediated PTEN deletion in the IgM-BCR expressing GCBs. All these results demonstrated that the PTEN expression level in *Pten^fl/fl^Cγ1^Cre/+^* mice was significantly impaired in GCBs. Thus, PTEN regulated AID transcription through PI3K-AKT signaling pathway in GCBs controls the CSR, IgG antibody response, and SHM.

## Discussion

We investigate the function of PTEN in regulating the strength of GC responses by employing a mouse model with the ablation of PTEN through Cγ1-Cre mediated recombination. Upon immunization, we found significantly higher IgM antibody responses and drastically lower IgG1 antibody responses in the *Pten^fl/fl^Cγ1^Cre/+^* mice compared to the control *Pten^+/+^Cγ1^Cre/+^* mice. Mechanistically, we found that the ablation of PTEN leads to the abnormal GC responses as demonstrated by: (i) severely disturbed compartmentalization of DZ and LZ; (ii) significantly decreased amount of IgG1-BCR expressing B cells; and (iii) the SHM in *Pten^fl/fl^Cγ1^Cre/+^* mice than the control *Pten^+/+^Cγ1^Cre/^*mice. Moreover, an *in vitro* CSR assay for the purified splenic B cells from *Pten^fl/fl^Cγ1^Cre/+^* mice also indicates the blunted CSR under a variety of differentiation-promoting conditions. Interestingly, the results of the impaired GC function in this report are different from the PTEN deletion in *Pten^fl/fl^CD19^Cre/+^, Pten^fl/fl^mb1^Cre/+^* or *Pten^fl/fl^* hCD20TamCre mice in the published studies (13, 16, 23), the deletion of *Pten^fl/fl^* loci in *Pten^fl/fl^Cγ1^Cre/+^* mice shall only be effective upon the transcription of *Cγ1-cre* gene, which shall be ideally only available in the IgG1-BCR expressing B cells. Thus, theoretically, only the IgG1 antibody responses shall be affected in *Pten^fl/fl^Cγ1^Cre/+^* mice upon immunization. However, an unexpected observation in this report is that the production of not only IgG1 but also IgG2b and IgG3 were significantly blunted in the *Pten^fl/fl^Cγ1^Cre/+^* mice. All these intriguing results were explained by the further mechanistic investigations show that LPS- and IL-4 stimulation robustly induced the transcription of Cγ1 in IgM-BCR expressing B cells, which efficiently disrupt the transcription of PTEN and AID in the stimulated splenic IgM-BCR expressing B cells from *Pten^fl/fl^Cγ1^Cre/+^* mice. There are eight sets of C_H_ exons organized as 5′-VDJ-Cμ-Cδ-Cγ3-Cγ1-Cγ2b-Cγ2a-Cε-Cα-3′ at the *Igh* locus in mice. Upon the CSR, the assembled V(D)J exons from Cμ encoded IgM-expressing naïve B cells is juxtaposed next to one of the sets of downstream C_H_ exons, allowing the production of different IgH classes (e.g., IgG3, IgG1, and IgG2b) (5). Thus, the transcription of Cγ1 in IgM-BCR expressing B cells and the subsequent disruption of PTEN and AID expression explained the poor production of not only IgG1 but also IgG2b and IgG3 in the *Pten^fl/fl^Cγ1^Cre/+^* mice. The observation of the pre-transcription of Cγ1 in IgM-BCR expressing B cells is consistent with the examination of the Cγ1 reporter mice, which reported the Cγ1 reporter gene expression in 85–95% of the GCB fraction 10–14 days after immunization with SRBC (33). Not a surprise, hyper-phosphorylated AKT and FoxO1 are observed as a result of the drastically reduced expression of PTEN. The reduced transcription of PTEN and AID are also confirmed by investigating the IgM-BCR expressing GCB cells *in vivo* from SRBC immunized *Pten^fl/fl^Cγ1^Cre/+^* mice. The hyper-phosphorylated AKT and FoxO1 in turn influence the AID expression in IgM-BCR expressing GCBs and reduce the differentiation of DZ GCBs partially through downregulation of the chemokine receptor CXCR4 (14, 15). However, whether or not the reduced expression of AID can directly contribute to the decrease in the DZ GCBs deserves further investigation. In the literature, the important functions of PI3K-AKT pathway on the regulation of cell growth, survival, proliferation, cell cycle and cellular metabolism were also reported (16, 19–22, 34, 35). Thus, it is also of interest to investigate how these events can influence the formation of GC structures.

In conclusion, our research provides an alternative mechanistic explanation for the significantly impaired CSR in PTEN deficient GCBs in addition to the recent published studies showing that constitutive PI3K activation or ablation of FOXO1 impairs AID targeting to particular switch regions. which leads to the partly lost CSR (14, 15). Our results demonstrate that PTEN regulated AID transcription in GCBs is essential for the CSR and IgG antibody response, and SHM.

## Ethics Statement

All animal protocols used in this study are approved by the IACUC (Institutional Animal Care and Use Committee) of Tsinghua University and performed in accordance with guidelines of the IACUC. The laboratory animal facility has been accredited by AAALAC (Association for Assessment and Accreditation of Laboratory Animal Care International). The assurance identification number is 15-LWL2 and was issued by Dr. Zai Chang, the vice chair of IACUC of Tsinghua University, Beijing, China.

## Author Contributions

WL and JW conceived, designed, and drafted the article; JW and SL performed experiments and laboratorial analysis; BH and MY supported the materials; JW and WL wrote the manuscript; WL, HQ, ZD, BH, MY, and JW reviewed and approved the manuscript final version.

## Conflict of Interest Statement

The authors declare that the research was conducted in the absence of any commercial or financial relationships that could be construed as a potential conflict of interest.
