# Supplementary material for: PTEN-Regulated AID Transcription in Germinal Center B Cells Is Essential for the Class-Switch Recombination and IgG Antibody Responses
Source: Front Immunol. 2018 Feb 28;9:371. doi: 10.3389/fimmu.2018.00371 (PMC5835858; doi:10.3389/fimmu.2018.00371)
Supplement: Supplementary file 1 [file Data_Sheet_1.pdf]

Figure S1

A

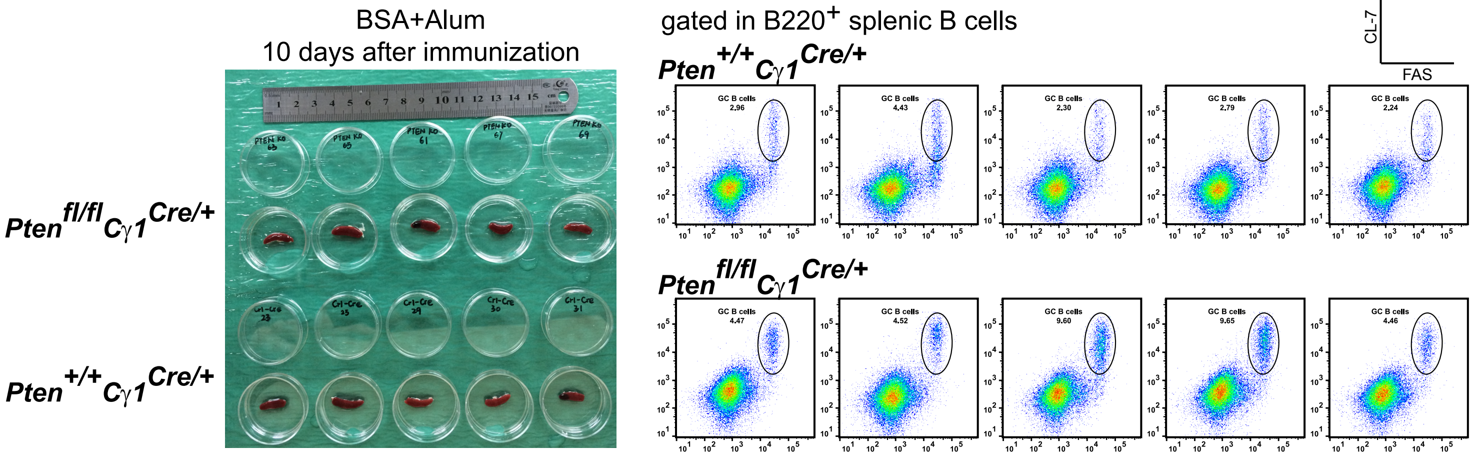
BSA+Alum
10 days after immunization
Pten fl/fl Cγ1 Cre/+
Pten +/+ Cγ1 Cre/+
gated in B220+ splenic B cells
CL-7
FAS
Pten +/+ Cγ1 Cre/+
GC B cells 2.96
GC B cells 4.43
GC B cells 2.30
GC B cells 2.79
GC B cells 2.24
Pten fl/fl Cγ1 Cre/+
GC B cells 4.47
GC B cells 4.52
GC B cells 9.60
GC B cells 9.65
GC B cells 4.46

B

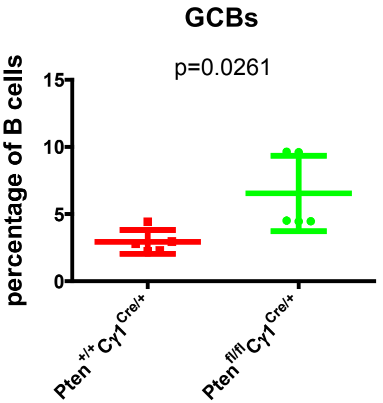
GCBs
p=0.0261
percentage of B cells
15
10
5
0
Pten +/+ Cγ1 Cre/+
Pten fl/fl Cγ1 Cre/+

Figure S1: Influenced germinal center formation in *Pten*$^{fl/fl}$ *Cγ1*$^{Cre/+}$ mice

(A) Macroscopic appearance of spleens from day 10 after the immunization in *Pten*$^{+/+}$ *Cγ1*$^{Cre/+}$ and *Pten*$^{fl/fl}$ *Cγ1*$^{Cre/+}$ mice by alum-precipitated BSA (left). Flow cytometry analysis of the increased GCBs number in the *Pten*$^{fl/fl}$ *Cγ1*$^{Cre/+}$ mice spleen (right). Values indicate the percentage of cells in the gated population. Data represented five mice per group in three independent experiments.

(B) The statistical quantification of flow cytometry data of germinal center (as described in A). Each symbol represents an individual animal; the data represent the mean ± SD in three independent experiments. Two-tailed *t* tests were performed for statistical comparisons.

Figure S2

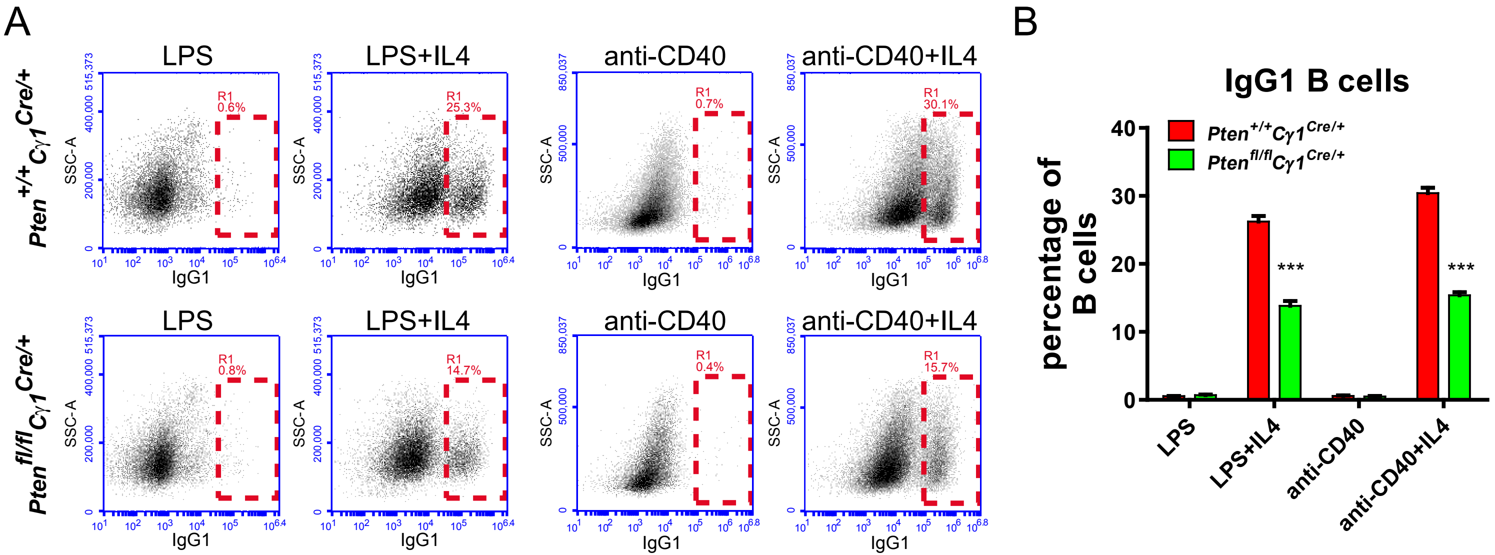
A
LPS
LPS+IL4
anti-CD40
anti-CD40+IL4
Pten+/+Cγ1Cre/+
Ptenfl/flCγ1Cre/+
SSC-A
IgG1
R1 0.6%
R1 25.3%
R1 0.7%
R1 30.1%
R1 0.8%
R1 14.7%
R1 0.4%
R1 15.7%
B
IgG1 B cells
percentage of B cells
Pten+/+Cγ1Cre/+
Ptenfl/flCγ1Cre/+
***
***
LPS
LPS+IL4
anti-CD40
anti-CD40+IL4

Figure S2: Damaged CSR in splenic B cells from *Pten$^{fl/fl}$ Cγ1$^{Cre/+}$* mice in an *in vitro* assay

(A) A defect in the class-switched IgG1-BCR expressing B cell population from *Pten$^{fl/fl}$ Cγ1$^{Cre/+}$* mice. Splenic B cells from 6-week-old *Pten$^{fl/fl}$ Cγ1$^{Cre/+}$* and control *Pten$^{+/+}$ Cγ1$^{Cre/+}$* mice were stimulated with LPS, LPS plus IL-4, anti-CD40, or anti-CD40 plus IL-4 for 4 days before flow cytometry analysis. The cells were stained with CD19 to pre-gated B cells and isotype-specific IgG1 to identify class-switched IgG1-BCR expressing B cells. The values indicate the percentage of cells in the gated population. Data were given from one representative of at least three independent experiments.

(B) The statistical quantification of the flow cytometry data of class-switched IgG1 as shown in (A). Data represent the mean ± SEM in three independent experiments. Two-tailed *t* tests were performed for statistical comparisons. ***, $p<0.001$.

Figure S3

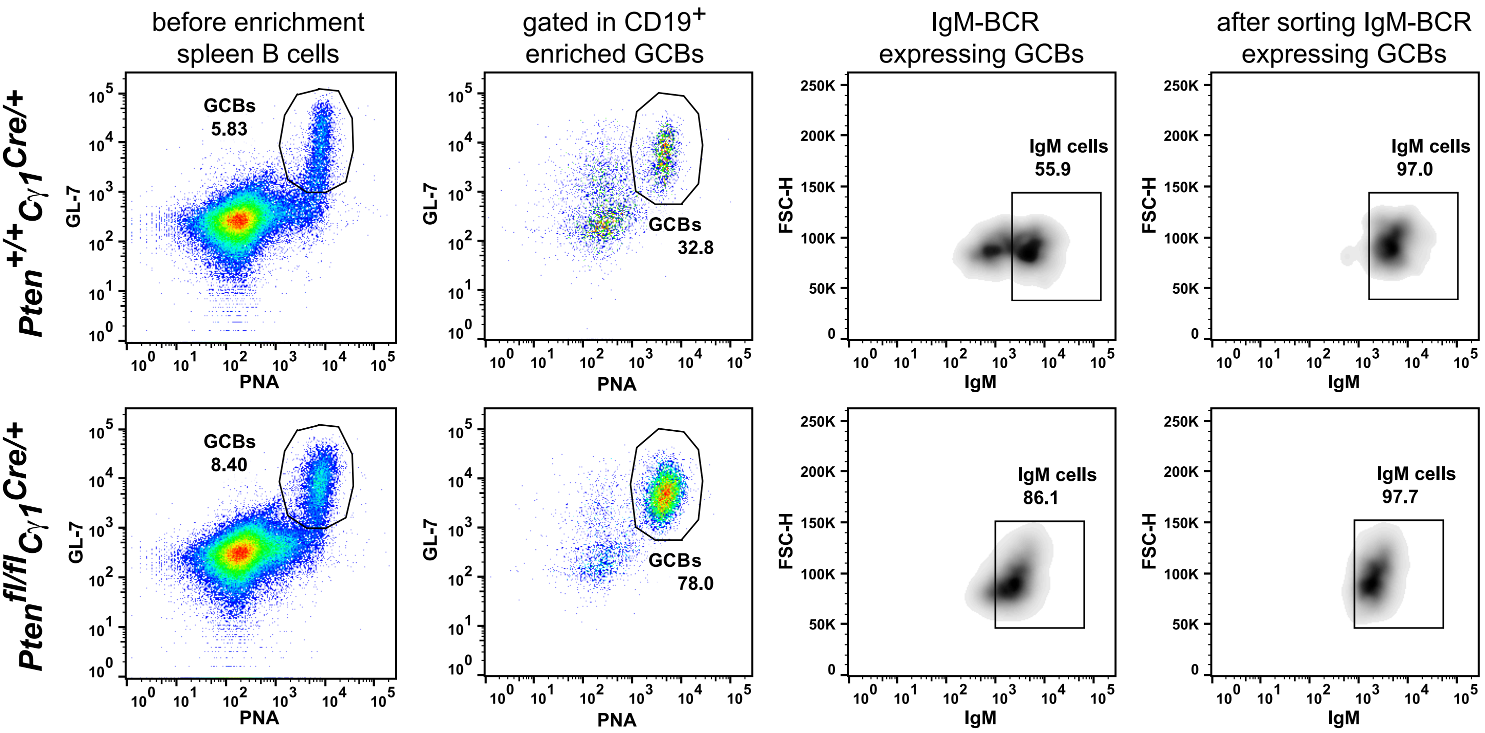

before enrichment
spleen B cells
gated in CD19+
enriched GCBs
IgM-BCR
expressing GCBs
after sorting IgM-BCR
expressing GCBs
Pten+/+Cγ1Cre/+
Ptenfl/flCγ1Cre/+
GCBs
5.83
GCBs
32.8
IgM cells
55.9
IgM cells
97.0
GCBs
8.40
GCBs
78.0
IgM cells
86.1
IgM cells
97.7
GL-7
PNA
FSC-H
IgM

Figure S3: Quality control for the sorting of IgM-BCR expressing GCBs

Flow cytometry sorting of IgM-BCR expressing GCBs from SRBC immunized *Pten*$^{+/+}$ *Cγ1*$^{Cre/+}$ and *Pten*$^{fl/fl}$ *Cγ1*$^{Cre/+}$ mice (4 mice for each group). Cells were sorted at day 7 after SRBC immunization. Values indicate the percentage of cells in the gated population.
